# Elucidation of a Causal Relationship Between Platelet Count and Hypertension: A Bi-Directional Mendelian Randomization Study

**DOI:** 10.3389/fcvm.2021.743075

**Published:** 2021-11-26

**Authors:** Po-Chun Chiu, Amrita Chattopadhyay, Meng-Chun Wu, Tzu-Hung Hsiao, Ching-Heng Lin, Tzu-Pin Lu

**Affiliations:** ^1^Department of Public Health, National Taiwan University, Taipei, Taiwan; ^2^Bioinformatics and Biostatistics Core, Center of Genomic and Precision Medicine, National Taiwan University, Taipei, Taiwan; ^3^Department of Medical Research, Taichung Veterans General Hospital, Taichung, Taiwan; ^4^Institute of Epidemiology and Preventive Medicine, National Taiwan University, Taipei, Taiwan

**Keywords:** Mendelian randomization, bi-directional causal estimation, hypertension, platelet count, Taiwan Biobank

## Abstract

Hypertension has been reported as a major risk factor for diseases such as cardiovascular disease, and associations between platelet activation and risk for hypertension are well-established. However, the exact nature of causality between them remains unclear. In this study, a bi-directional Mendelian randomization (MR) analysis was conducted on 15,996 healthy Taiwanese individuals aged between 30 and 70 years from the Taiwan Biobank, recorded between 2008 and 2015. The inverse variance weighted (IVW) method was applied to determine the causal relationship between platelet count and hypertension with single nucleotide polymorphisms as instrumental variables (IVs). Furthermore, to check for pleiotropy and validity of the IVs, sensitivity analyses were performed using the MR-Egger, weighted median and simple median methods. This study provided evidence in support of a positive causal effect of platelet count on the risk of hypertension (odds ratio: 1.149, 95% confidence interval: 1.131–1.578, *P* < 0.05), using the weighted median method. A significant causal effect of platelet count on hypertension was observed using the IVW method. No pleiotropy was observed. The causal effect of hypertension on platelet count was found to be non-significant. Therefore, the findings from this study provide evidence that higher platelet count may have a significant causal effect on the elevated risk of hypertension for the general population of Taiwan.

## Introduction

Over the past two decades, hypertension has been reported as a major risk factor for diseases such cardiovascular disease (CVD), diabetes, and kidney disease (1). Hypertension has high incidence and prevalence in developing countries all over the world (2). The Global Burden of Disease study, a collaboration between the World Health Organization and the World Bank, reported detailed risk factors for blood pressure using disability-adjusted life-years, a metric calculated based on premature death due to heart disease (3, 4). Furthermore, recent surveys have reported an increasing prevalence of hypertension in Taiwan, with ~25% of the population being affected (5), and stroke, diabetes mellitus, and CVD are among the leading causes of overall mortality in the Taiwanese population (6). Taiwan has been active in trying to control hypertension, because of its ramifications on public health, and the Taiwan Hypertension Society and the Taiwan Society of Cardiology have been jointly devising policies and methods to monitor hypertension (5). However, not much information is available on the causes of hypertension, and studies designed to understand the factors that have a causal effect on hypertension are needed.

Hypertension is a multi-factorial disease (7–9) that has a strong correlation with a person's platelet count (10). Moreover, American and European clinical guidelines propose antiplatelet drug treatments for reduction of the risk of CVD in patients (11). Similarly, antihypertensive therapies also decrease and prevent the risk of CVD (12, 13). Thus, it can be hypothesized that there exists an association between platelet count and risk of hypertension (14, 15). However, if an exposure has a non-causal association with an outcome, then treatments targeted at the exposure will likely have no real benefit. Therefore, identifying potential risk factors for hypertension and establishing a causal relationship is an emergent public health issue.

Mendelian randomization (MR) studies assess causal inference of a modifiable exposure on an outcome by using genetic alleles as unbiased instruments (16). Observational studies fail to provide conclusive inferences regarding causality, as confounding effects and reverse causality may bias the exposures, thereby leading to overestimation of associations. MR studies are based on the assumption of random assortment of genetic alleles during meiosis and can confer advantages similar to that of a randomized controlled trial by investigating the relationship between genetic alleles that are exclusively associated with an exposure and that affect the disease risk only through exposure (17). The resulting determination of causality is thus free from the effects of confounding and reverse causality. That being said, determining causality alone is not sufficient. The direction of the causality, i.e., whether a rise in platelet counts precedes hypertension or whether hypertension causes elevation of platelet counts, is important for understanding clinical repercussions, which cannot be inferred from simple regression analysis. Therefore, this study aimed to elucidate the bi-directional causality of platelet counts and hypertension using single nucleotide polymorphisms (SNPs) as the instrumental variables (IVs) in a one-sample MR setting, using ~16,000 healthy Taiwanese participants from the Taiwan Biobank (TWB) database.

## Methods

### Data Source

TWB is an extensive community-based database that facilitates large-scale cohort studies and case-control studies on various diseases by combining genetic and clinical information from healthy volunteers and patients from Taiwan (18). It has recruited participants between 30 and 70 years of age with no history of cancer. The hospital-based component has recruited patients affected by the most common chronic diseases in Taiwan, including CVD, diabetes, chronic kidney disease, and so on. This study includes a total of 15,996 Taiwanese subjects of Han-Chinese ancestry (randomly selected from 2008 to 2015) from TWB that were genotyped using the Axiom-Taiwan Biobank Array Plate (TWB chip; Affymetrix Inc, CA, USA). The genotype data consist of a total of 653,291 gene variant sites and 646,735 SNPs. The TWB study was approved by the ethics committee at Taichung Veterans General Hospital (IRB: TCVGHNo.CE16270B-2). Consent was not obtained because the data were de-identified.

### Study Population

As the data included in this study were recorded between 2008 and 2015, the contemporaneous definition of hypertension from the American Heart Association (https://www.heart.org/) was adhered to, and three inclusion criteria were adopted: (i) average sitting systolic blood pressure ≥ 140 mmHg, (ii) average sitting diastolic blood pressure ≥ 90 mmHg, and/or (iii) self-report of hypertension *via* questionnaires. The platelet count at the baseline measurement in TWB in units of 10^3^/μL was included as the exposure variable in the study. The normal range of platelet count is 150–500 10^3^/μL, and the number is susceptible to the influence of external factors (19).

### Exclusion Criteria

Individual quality control and SNP quality control were conducted. To maintain high data quality and avoid spurious associations, individuals with missing genotype >5% and SNPs with call rate <97% were removed from the study. A cryptic relatedness check was conducted among individuals, to ensure no first, second, or third-degree relatives were included in the analysis. A sex check was done to confirm there were no anomalies present in the reported sex of the study individuals. Hardy-Weinberg equilibrium was ensured for all SNPs. Finally, pairwise linkage disequilibrium (LD) for whole genome SNPs was calculated, and those with high LD (r^2^ > 0.8) were pruned out. All quality control steps were carried out by PLINK1.9, beta version (20).

### Statistical Analysis

#### Mendelian Randomization

**Figure 1:**
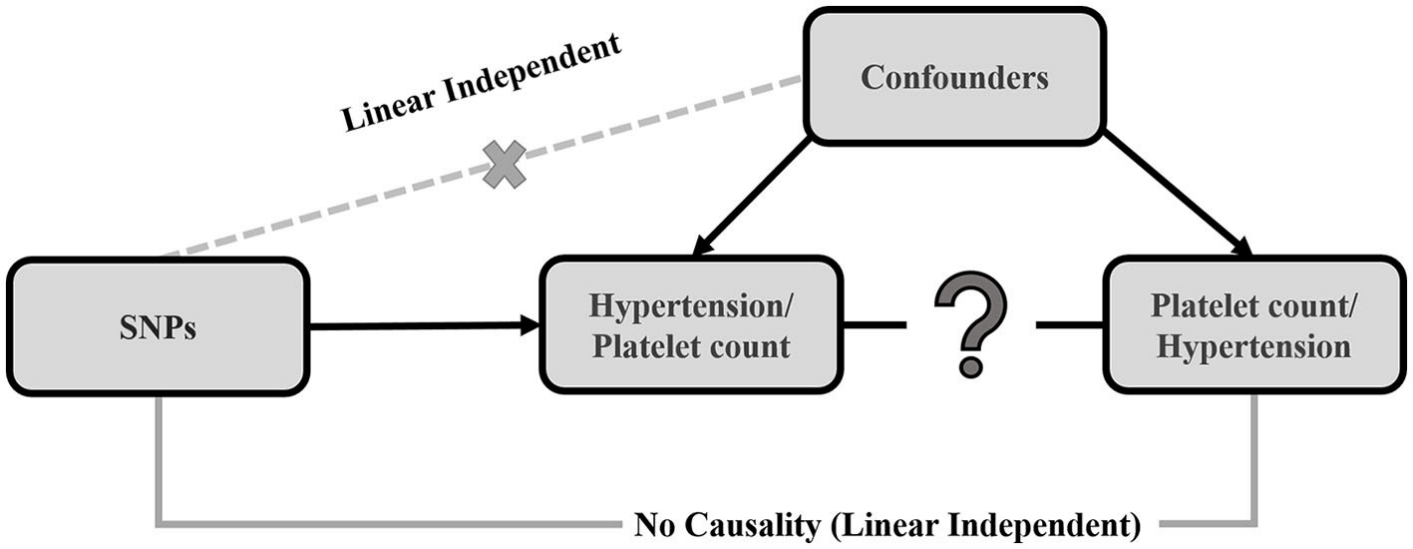
Causal directed acyclic graph, displaying the 3 assumptions of Mendelian randomization. The black arrows show instrumental variable (IV) assumptions. The crossed dotted gray lines depict the violations of the assumptions. The single genetic variant (SNP) that satisfies the IV assumption is *G*_*j*_. The effect of the single genetic variant (SNP) on the exposure (X) is β_*xj*_ and the causal effect of the exposure (X) on the outcome (Y) is Θ.

The MR method was applied to determine the causal relationship between platelet count and hypertension using SNPs as the IVs (21–23). The three essential assumptions that are prerequisites for conducting MR are: (a) the relevance assumption, the IV is predictive of the exposure; (b) the independence assumption, the IV is independent of any confounding factors of the exposure-outcome association; and (c) the exclusion-restriction assumption, the IV is conditionally independent of the outcome given the exposure and the confounding factors (24). Each of these assumptions were true in the present case. The inverse variance weighted (IVW) method was used in this study to elucidate the causality between platelet count and hypertension (25). The selected genetic variants can only affect the outcome *via* the risk factor if they meet the above conditions (22, 26). Figure 1 displays a directed acyclic graph showing the MR assumptions for valid causality of exposure and outcome. Finally, the MR-Egger method (27) and the simple and weighted median methods (28) were used to conduct sensitivity analyses. All *P*-values reported in this study are 2-sided.

#### Instrument Variables

The genome-wide association summary statistics for platelet count and hypertension was evaluated by PLINK2.0 linear regression and logistic regression association analysis (29). SNPs with genome-wide significance (*P* < 5e-6) were selected as instruments for the analysis. The first assumption of relevance was thus empirically met. To justify the independence and exclusion-restriction assumptions, sensitivity analysis (MR-Egger, weighted median) was conducted, thereby establishing the validity of the IVs. The F-statistic can reflect the exact strength of the effect of SNPs on the exposure traits. F-statistics were evaluated by a Gaussian regression model of the genetic components and the exposure trait after adjusting for all confounders (30). A threshold of *F*-statistic ≥10 has been proposed for MR analysis to avoid weak instrument bias (31). Therefore, weak instrument bias was eliminated by excluding instruments where the F-statistic was <10.

#### Inverse Variance Weighting

The association of the IV with exposure $(\hat{\beta_{xj)}}$ and outcome $(\hat{\beta_{Yj})}$ variables can be obtained by regressing exposure and outcome on jth IV (genetic variant *G*_*j*_). Consequently, the causal effect of the exposure on the outcome can be estimated by $\hat{\theta_{j}}=\frac{\hat{\beta_{Yj}}}{\hat{\beta_{Xj}}}$. The IVW method estimates the overall causality when multiple IVs exist by averaging over all causal estimates for each of the IVs (32). The workability of the IVW method rests on the assumption that the IVs are valid, that is, they are uncorrelated and do not display pleiotropy. Given that there exists no correlation among the IVs, the IVW estimate asymptotically tends toward a two-stage least squares causal estimate. The causal estimate can be mathematically expressed as (26, 33).


$${\hat{\theta }}_{IVW}=\frac{\sum_{j}{\hat{\beta }}_{Yj}{\hat{\beta }}_{Xj}se{\left({\hat{\beta }}_{YJ}\right)}^{2}}{\sum_{j}{\hat{\beta^{2}}}_{Xj}se{\left({\hat{\beta }}_{YJ}\right)}^{2}}$$

where ${\hat{\beta }}_{Yj}=\theta_{IVW}{\hat{\beta }}_{Xj}+\epsilon_{ij};\;\epsilon_{ij}\sim N\;\left(0,\sigma^{2}se{\left({\hat{\beta }}_{Yj}\right)}^{2}\;\right)$.

#### Pleiotropy of Instrumental Variables

**Figure 2:**
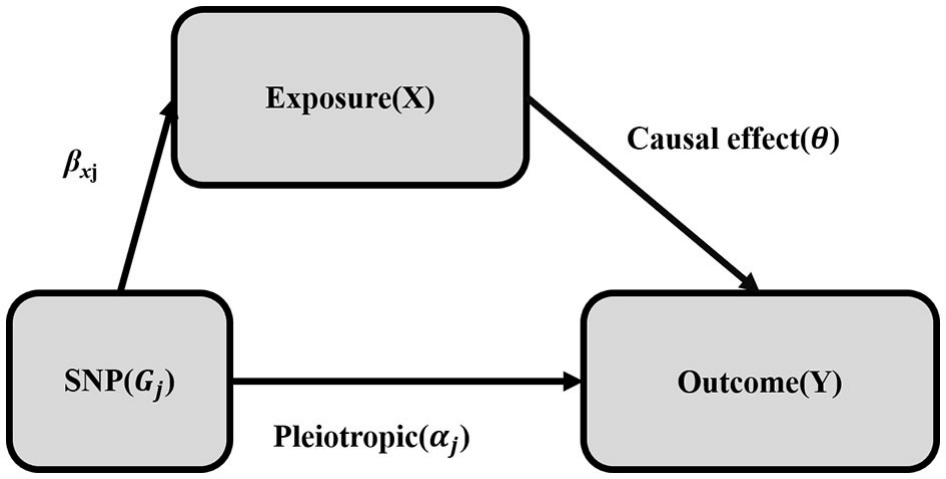
Mendelian randomization with pleiotropy. The association of the genetic variant (SNP) with the outcome (Y) can be decomposed into the indirect effect *via* exposure (X) (Mendelian randomization: exclusion-restriction assumption) and the direct (pleiotropic) effect. The single genetic variant (SNP) that satisfies the instrumental variable (IV) assumption is *G*_*j*_. The effect of the IV (SNP) on the exposure (X) is β_xj_, the direct (pleiotropic) effect on the outcome (Y) is α_j_ and the causal effect of the exposure (X) on the outcome (Y) is θ.

IVW is a naïve MR method that assumes validity of all genetic variants as IVs. However, the genetic variants may be invalidated by a phenomenon-called pleiotropy, where the genetic variants may be associated with more than one phenotype. For instance, the genetic variants might have an association with the outcome of the study through an alternate pathway (34). Mathematically this can be conceived as β_*Yj*_ = α_*j*_ + θβ_*Xj*_, where the association of the genetic variants and the outcome (β_*Yj*_) is the result of gene pleiotropy (α_*j*_) plus the indirect causal effect of genetic variants on the outcome *via* other risk factors (β_*Xj*_) (Figure 2). Therefore, to confirm any causality that is observed using the IVW method, sensitivity analyses such as the MR-Egger method and the simple and weighted median methods were implemented.

#### Sensitivity Analysis

##### MR-Egger Method

The MR-Egger method again uses summarized genetic data to conduct MR, and can not only estimate the pleiotropic effects as part of the analysis but can also determine the direction of the pleiotropy. The MR-Egger method should conform to the InSIDE assumption (Instrument Strength Independent of Direct Effect), which is that the pleiotropic effects α_*j*_ are distributed independently from the association of the genetic variants with the exposure (35). The regression model can be written as:


$$\begin{array}{l} {\hat{\beta }}_{Yj}=\alpha_{j}+\theta \beta_{Xj}+\epsilon_{Ej};\;\epsilon_{Ej}\sim N\left(0,{\sigma^{\prime }}^{2}se{\left({\hat{\beta }}_{Yj}\right)}^{2}\right) \end{array}$$

where α_*j*_ is the intercept and θ is the slope (36). The regression equation for MR-Egger differs from IVW by the intercept term α_*j*_. If pleiotropy is balanced (α_*j*_ = 0) then the IVW method consistently provides an estimate of the causality, whereas if α_*j*_ ≠ 0, then there either exists directional pleiotropy and/or the InSIDE assumption is violated. Hence, the MR-Egger intercept test confirms the validity of the IVs, where a non-zero intercept indicates that the causal inference from IVW may have some bias.

##### Weighted Median Method

Compared to the IVW and MR-Egger methods, the weighted median method has greater robustness to individual genetic variants with strong outlying causal estimates. It calculates the median of the ratio of IV estimates evaluated using each genetic variant individually. The simple median method gives a consistent estimate of the causal effect with at least 50% of the genetic variants as valid IVs. For the weighted median method, 50% of the weight comes from valid IVs. Also, it will not be affected by outliers and high-leverage genetic variants (28).

## Results

### Inclusions and Exclusions

**Figure 3:**
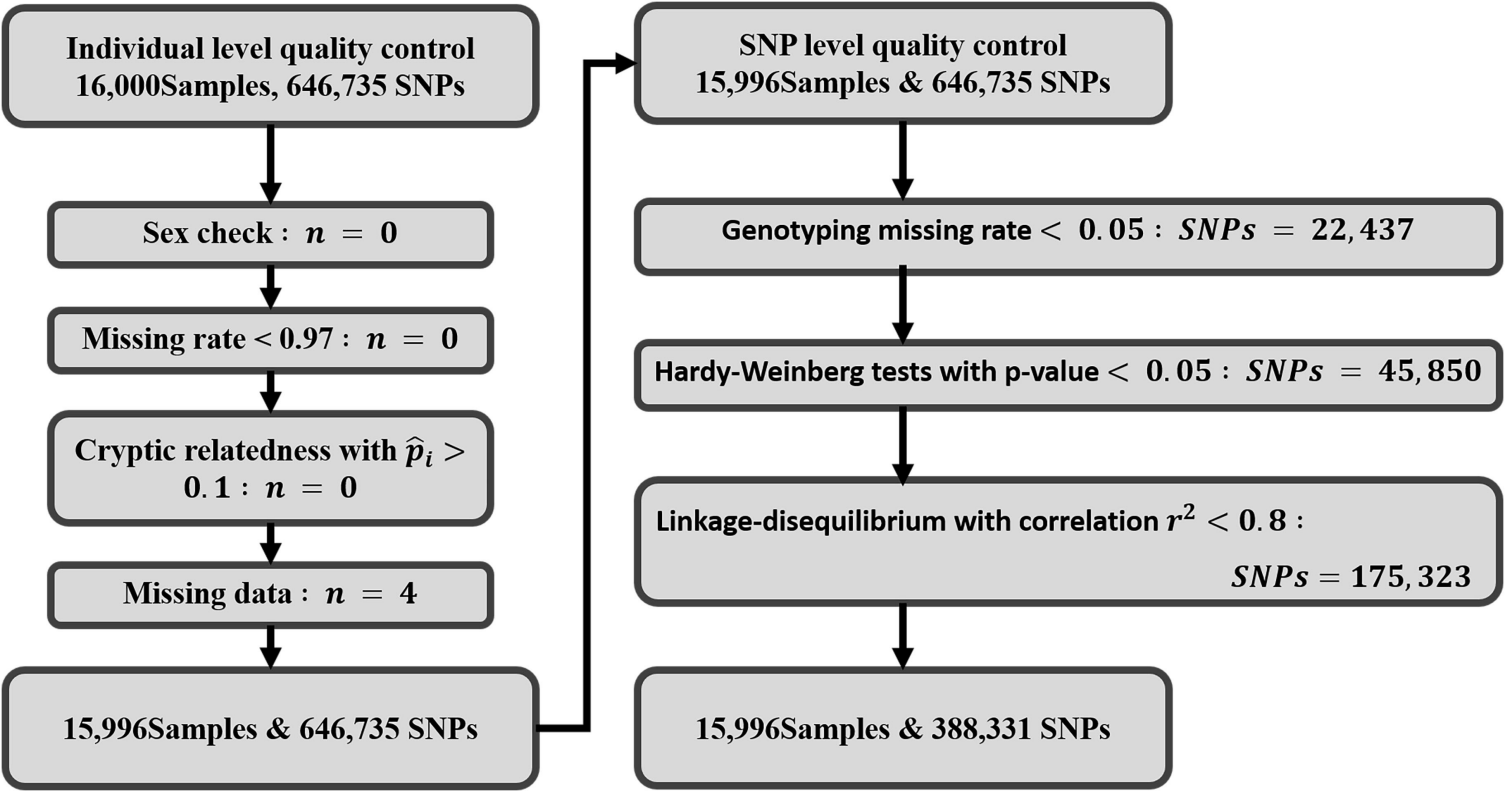
Work flow displaying the quality control steps: exclusion criteria and inclusions of SNPs and individuals. A total of 16,000 samples and 646,735 SNPs were initially retrieved from TWB for this study. After quality control and exclusions, 15,996 Taiwanese subjects and 388,331 SNPs were retained for final analysis.

Figure 3 gives an overview of the exclusions and inclusions of this study. Four subjects with missing data on platelet count were excluded from the study. For individual-level quality control, no subjects were removed due to missing genotype, high cryptic relatedness, or sex anomalies. A total of 646,735 autosomal SNPs underwent SNP-level quality checks where 22,437 variants were excluded with call rate <97%, 45,850 variants were removed that deviated from Hardy-Weinberg equilibrium, and 175,323 variants were pruned due to pairwise LD. Finally, a total of 15,996 participants and 388,331 variants were included in the study for further analysis.

### Study Participants

**Figure 4:**
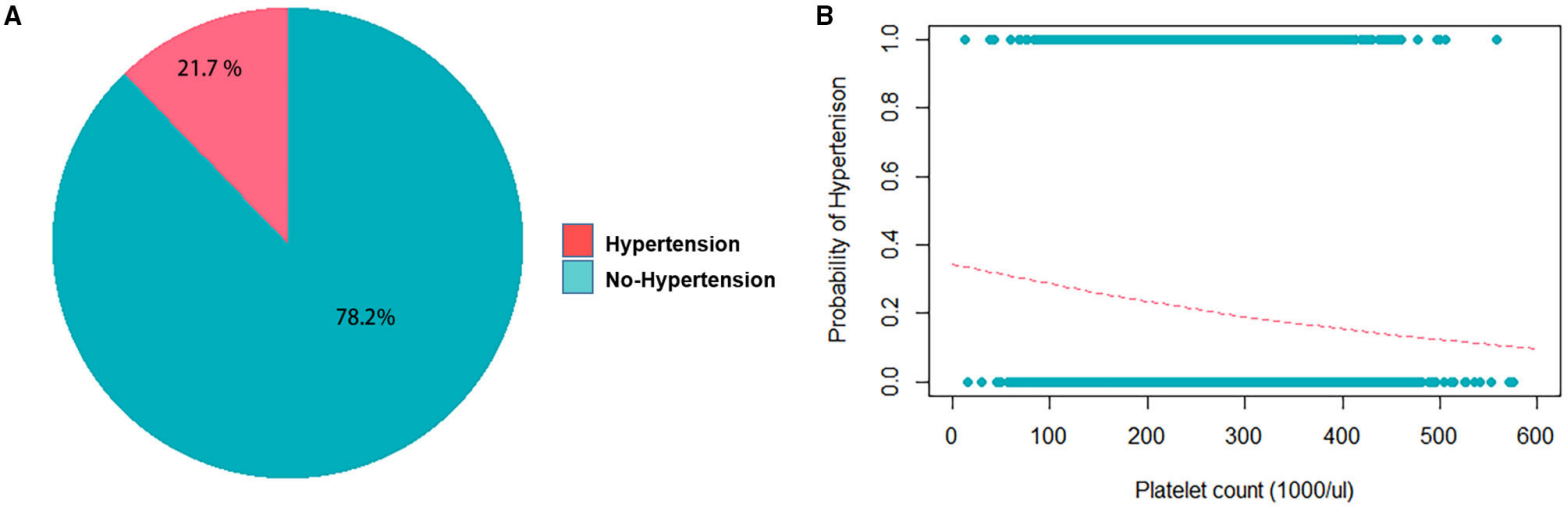
Frequency distribution of hypertension and platelet counts in the study population. **(A)** Pie chart displaying fractions of the study population with and without hypertension. **(B)** Logistic regression without adjustment of confounders shows the probability of getting hypertension when the platelet count increases. The coefficient of the platelet count is −0.0026716 with the *P* = 1.85e-14.

The average age of our study population was 48.7 years, and 49.8% of the subjects were male (Table 1). Based on the definition of the American Heart Association, 3,480 patients in this study (21.8%) were classified as hypertensive (Table 1, Figure 4A). This percentage was a little lower than prior reports from Taiwan (37), as this study was conducted on a healthy population from TWB. Platelet count in the study participants had a mean ± SD of 231.1 ± 57.79 10^3^/μ*L* (Table 1) among hypertensive patients. Table 1 lists the other clinical characteristics that are known risk factors for hypertension. A threshold of >240 (1,000/μL) was determined as a high platelet count, through ROC analysis using both sex-age adjusted and multivariate adjusted models (Supplementary Figure S1).

**Table 1:** Characteristics of study participants from the Taiwan Biobank database.

	**Hypertension**	**Non-hypertension**	***P-*value**
	***n* = 3,480**	***n* = 12,516**	
Sex			<2 × 10^−16^
Female	1,284 (36.9%)	6,747 (53.9%)	
Male	2,196 (63.1%)	5,769 (46.1%)	
Age (years)	56.52 ± 9.62	47.82 ± 11.07	<2 × 10^−16^
Platelet count (10^3^/μL)	231.1 ± 57.79	239.49 ± 56.55	1.85 × 10^−14^
Fasting glucose (mg/dL)	103.2 ± 26.17	94.65 ± 18.93	<2 × 10^−16^
Hematocrit (%)	44.44 ± 4.40	43.42 ± 4.57	<2 × 10^−16^
Triglyceride (mg/dL)	140.2 ± 91.70	111.16 ± 91.28	<2 × 10^−16^
High-density lipoprotein cholesterol (mg/dL)	49.62 ± 12.15	54.10 ± 13.20	<2 × 10^−16^
Hemoglobin (g/dL)	14.1 ± 1.49	13.91 ± 1.58	<2 × 10^−16^
Red blood cell count (MILON/μL)	4.89 ± 0.53	4.78 ± 0.52	<2 × 10^−16^
White blood cell count (10^3^/μL)	6.34 ± 1.64	6.03 ± 1.56	<2 × 10^−16^

*Data are presented as mean ± SD unless otherwise indicated. Study includes Taiwanese subjects, of Han-Chinese ancestry, randomly selected from 2008 to 2015, from Taiwan Biobank*.

### Statistical Analysis

Regression analyses were conducted to test the crude association between platelet count and hypertension using the study dataset. First, a logistic regression was used to assess platelet count as the exposure and hypertension as the outcome (binary) (Figure 4B), followed by an analysis of the reverse association using a linear regression analysis, with hypertension as the exposure and platelet count as the outcome (continuous). Table 2 displays the results of the logistic regression that was conducted with hypertension as the outcome, and platelet count was found to be significantly associated (*P* < 0.05) when adjusted for sex, age, fasting glucose, hematocrit, triglycerides, high-density lipoprotein cholesterol, hemoglobin, red blood cell count, and white blood cell count. In the linear regression with platelet count as the outcome, hypertension was found to be significantly associated with platelet count (*P* < 0.05) when adjusted by the same confounders (Table 3).

**Table 2:** Logistic regression analysis results, with hypertension as outcome and platelet count as exposure/risk factor, and with known confounders adjusted.

	**Beta**	**Standard error**	***P-*value**
Platelet count	1.21e-03	5.99e-05	<0.05
Sex (reference = male)	−5.13e-02	8.50e-03	<0.001
Age	1.14e-02	2.83e-04	<0.001
Fasting glucose	1.32e-03	1.52-04	<0.001
Hematocrit	−6.13	1.42e-03	<0.001
Triglyceride	1.70e-04	3.71e-05	<0.001
High-density lipoprotein cholesterol	−1.96e-03	2.72e-04	<0.001
Hemoglobin	2.07e-02	4.26e-03	<0.001
Red blood cell count	3.02e-02	7.47e-03	<0.001
White blood cell count	1.86e-02	2.09e03	<0.001

**Table 3:** Linear regression results with platelet count as outcome, hypertension as exposure/risk factor, and with known confounders adjusted.

	**Beta**	**Standard error**	***P-*value**
Hypertension	2.11	1.04	<0.05
Sex (female vs. male)	14.02	1.12	<0.001
Age	−1.02	0.04	<0.001
Fasting glucose	0.03	0.02	0.14
Hematocrit	−1.67		<0.001
Triglyceride	0.03	0.01	<0.001
High-density lipoprotein cholesterol	−0.01	0.04	0.80
Hemoglobin	−5.09	0.56	<0.001
Red blood cell count	5.70	0.99	<0.001
White blood cell count	10.70	0.26	<0.001

*Study conducted on Taiwanese subjects, of Han-Chinese ancestry, randomly selected from 2008 to 2015, from Taiwan Biobank*.

#### Causal Effect of Platelet Count on Hypertension

Over the last decade, many genome-wide association studies (GWASs) with sample sizes >10,000 have reported 5 SNPs (rs385893 in *JAK2*, rs11082304 in *CABLES1*, rs6425521 in *DNM3*, rs4895441 in *HMIP*, and rs7775698 in *HBS1L*) that are associated with platelet count (Supplementary Table S1). A GWAS was conducted to determine the association of SNPs with platelet count (*P* < 5e-6) using TWB, and an F-statistic threshold of ≥10 was applied to eliminate those with weak instrument bias. All 5 of the previously reported variants were selected (Table 4) as instruments. One-sample MR for the causal inference of platelet count (exposure) on hypertension (outcome), with the 5 variants as IVs, was conducted using different methods as shown in Table 5. All 5 variants conformed to the three basic assumptions, to be considered as valid instruments in the analysis. Sex, age, fasting glucose, hematocrit, triglycerides, high-density lipoprotein cholesterol, hemoglobin, red blood cell count, and white blood cell count were used as confounders. A significant positive causal association was observed by IVW as well as by the simple median and weighted median methods. A positive causal association of platelet count with the risk of hypertension (odds ratio 1.149, 95% confidence interval 1.131–1.578, *P* < 0.05) was observed using the weighted median method. However, no significant causal effect was observed when the MR-Egger method was used. Furthermore, the intercept observed with the MR-Egger method was not significant, implying that the 5 SNPs related to platelet count did not display any pleiotropic effects.

**Table 4:** List of SNPs with genome-wide significance using Taiwan Biobank data.

**SNP**	**Gene**	**CHR**	**Beta**	***P*-value**
**(a) SNPs significantly associated with platelet count**				
rs6425521	DNM3	1	4.42	5e-06
rs7775698	HBS1L	6	8.27	5e-06
rs4895441	HMIP	6	7.34	5e-06
rs385893	JAK2	9	4.99	5e-06
rs11082304	CABLES1	18	3.2	5e-06
**(b) SNPs significantly associated with hypertension**				
rs1458038	FGF5	4	1.197	5e-08
rs3796605	FGF5	4	0.86	5e-08
rs455938	MAST4	5	1.15	5e-08
rs10866754	CTC-535M15.2	5	1.17	5e-08
rs648435	APHGAP42	11	0.86	5e-08
rs2018159	APHGAP42	11	0.86	5e-08

*All SNPs reported here reached the threshold for significance: P <5e-06. (a) Linear regression analysis of platelet count on SNPs, adjusted by sex, age, known confounders, and the top 10 principal components from principal components analysis (PCA) conducted on the genotype data. (b) Logistic regression analysis of hypertension on SNPs, adjusted by sex, age, known confounders, and the top 10 principal components from PCA conducted on the genotype data. The study was conducted on Taiwanese subjects of Han-Chinese ancestry, randomly selected from 2008 to 2015, from Taiwan Biobank*.

**Table 5:** Bi-directional causality of platelet count and hypertension.

**Method**	**Estimate**	**Standard error**	**95%CI**	***P*-value**
**Hypertension as outcome and platelet count as exposure**				
IVW	0.121	0.061	[0.001–0.240]	0.049
Simple median	0.139	0.012	[0.115–0.162]	<0.0001
Weighted median	0.134	0.009	[0.116–0.152]	<0.0001
MR-Egger	0.048	0.208	[−0.358–0.455]	0.816
(Intercept)	0.444	1.206	[−1.919–2.807]	0.713
**Platelet count as outcome and hypertension as exposure**				
IVW	0.343	0.258	[−0.164–0.849]	0.185
Simple median	0.446	0.315	[−0.171–1.063]	0.156
Weighted median	0.254	0.316	[−0.366–0.874]	0.423
MR-Egger	−1.294	0.694	[−4.613–2.025]	0.445
(Intercept)	1.703	1.714	[−1.710–5.166]	0.328

*IVW, inverse variance weighting; MR, Mendelian randomization. Sex, age, fasting glucose, hematocrit, triglycerides, high-density lipoprotein cholesterol, hemoglobin, red blood cell count, and white blood cell count were used as confounders. The study was conducted on Taiwanese subjects of Han-Chinese ancestry, randomly selected from 2008 to 2015, from Taiwan Biobank*.

#### Causal Effect of Hypertension on Platelet Count

Supplementary Table S2 lists, from prior studies, SNPs that were significantly associated with hypertension and had an F-statistic ≥10. In this study, a GWAS using TWB data to test the association of SNPs with hypertension reported 6 SNPs (rs1458038 in *FGF5*, rs3796605 in *FGF5*, rs455938 in *MAST4*, rs10866754 in *CTC*-535M15.2, rs648435 in *APHGAP42*, and rs2018159 in *APHGAP42*) to be significantly associated (*P* < 5e-6) with hypertension (Table 4). A one-sample MR for the causality of hypertension (exposure) on platelet count (outcome) was conducted, with these 6 SNPs as IVs, using the methods described above (Table 5). The 6 variants were considered valid instruments as they met the three basic assumptions. Again, sex, age, fasting glucose, hematocrit, triglycerides, high-density lipoprotein cholesterol, hemoglobin, red blood cell count, and white blood cell count were used as confounders in the analyses. None of the methods showed a significant causal effect of hypertension on platelet count, and the intercept from the MR-Egger method was also found to be non-significant, suggesting that the 6 SNPs related to hypertension did not display any pleiotropy. Both IVW and the median-based methods indicate that the weighted causal effect is positive but not significant (Table 5).

## Discussion

With hypertension being a serious risk factor for conditions such as CVD, diabetes mellitus, kidney disease, and stroke, detecting elevated levels of blood pressure early on can lead to prevention and control of hypertension. Moreover, several factors such as urbanization, an aging population (high life expectancy), smoking, drinking alcohol, a sedentary lifestyle, and so on, are related to a higher incidence of hypertension (4). Therefore, hypertension has a become a public health issue. Furthermore, >50% of hypertensive individuals are not aware of the condition. Population-based studies, with the goal of identifying causal factors for hypertension, can assist in its early detection, and therefore can help prevent mortality due to CVD. In this study, we conducted a bi-directional causality study for hypertension and platelet count using a general population from Taiwan. A plethora of studies exist in the literature that discuss the elevated platelet counts in CVD patients (38–40). Our study showed that even though both hypertension and platelet count displayed significant association, higher platelet count was observed to have a causal effect on the elevated risk of hypertension.

The prevalence of hypertension rises with age in both males and females; however, rates of developing hypertension in premenopausal women are lower than that of men of the same age (41) while after the age of 50, women apparently have higher rates of hypertension than men (42). Similarly, platelet count has been shown to be a genetic trait regulated by both sex and age (43). Lower platelet counts were observed in men than in women, and platelet count was observed to decrease with age (Supplementary Figure S2). Both age and sex appear to be factors that have significant effects on hypertension and platelet count, and this was reiterated in our analysis (Tables 2, 3). Thus, they were used as covariates for adjustment in our bidirectional MR analysis. Regardless of sex, all people with hypertension are at an increased risk of developing CVD (43). Furthermore, platelet disorders such as thrombocytosis, where the body releases too many platelets, can be triggered by diseases/conditions such as acute bleeding and blood loss, cancer, infections, iron deficiency, splenectomy, and hemolytic anemia (44), while conditions such as alcoholism, autoimmune diseases, bone marrow diseases, and cancer treatments like chemotherapy and radiation therapy can cause thrombocytopenia (low platelet count disorder) (45). However, hypertension has scarcely been reported as a cause for platelet disorders in prior studies. On the other hand, many studies have reported numerous platelet abnormalities including morphologic, biochemical, and functional, to have a causal association with hypertension (10, 14, 46, 47). Our MR analysis confirms all of the above.

Hypertensive drugs may potentially reverse platelet changes, which may occur due to either the direct effect of the antihypertensive drug on the platelets or due to the indirect effect of lowered blood pressure as a result of reduced stress and improved endothelial cell function (47). The effects of antihypertensive drugs on platelet function have been thoroughly characterized through both *in vitro* experiments and clinical studies. Findings have not been consistent for a given drug class, showing different effects on different parameters of platelet function. In addition, given the wide diversity of study designs, study subjects, and methodologies, it is not yet known if one specific class of drug works better than the others. Although no information about antihypertensive medication use is available in the TWB data, the major conclusion of this study stands, as prior studies have shown consistent findings even after excluding patients who are on antihypertensive drugs (10). Also, there was essentially no reporting bias in this study. This is because among the 3,480 hypertensive patients analyzed in this study, 2,052 were identified based on self-reported questionnaires and 1,428 were identified based on blood pressure measurements. Therefore, patients who took antihypertensive drugs were not classified as healthy, but were identified as hypertensive based on the questionnaire. Hence, these subjects were not neglected due to the treatment effects of drugs.

In hypertension, activation of platelets and endothelial cells leads to thrombotic tendency, which may further result in CVD events such as myocardial infarction and stroke. However, the full pathophysiological mechanism of hypertension has yet to be defined. Hypertension arises due to compromised balance between vasodilators and vasoconstrictors, leading to changes in the vascular structure that result in high blood pressure (48). Elevated platelet counts result in either excessive clotting or abnormal bleeding (49). Prior studies have shown that SNPs associated with platelets may alter platelets in subtle ways to raise the risk of CVD in combination with other risk factors (49). This is precisely what MR analysis illustrates. It infers the causality of an IV-induced modifiable exposure on the outcome. Hence, we believe the SNPs chosen as instruments in the current study had a modifiable effect on the platelet counts, thus demonstrating a positive causality of high platelet count on the risk of developing hypertension. Platelets are blood cells in plasma that stop bleeding by sticking together to form a clot. High platelet counts may cause spontaneous and sudden development of blood clots, and abnormal blood clotting can lead to fatal conditions such as stroke and heart attack. Therefore, based on our findings, higher platelet counts could be used as an early risk indicator of future cardiac events and can be used for public health prevention. Other hematological parameters such as white blood cell count and hematocrits are established prognostic markers of hypertension and CVD and have been shown to be significantly associated through our analysis, indicating that the association may have been on a bone marrow level (50). However, as this was out of the scope of this study, no further analysis was conducted using hematological parameters.

As described above, the validity of IVs in MR depends on 3 assumptions: relevance, independence, and exclusion-restriction. However, only the first assumption can be fully empirically tested, because the second and third assumptions depend on all possible confounders of the exposure-outcome association, both measured and unmeasured. For the IVW method, all of the genetic variants included in the study as IVs need to satisfy the MR assumptions to elucidate a consistent estimate of the causal effect (28). To confirm this, the MR-Egger method and the simple and weighted median methods were conducted as sensitivity analyses. The MR-Egger method estimates the true causal effect consistently under a weaker assumption known as InSIDE, even when all genetic effects are invalid due to violation of the third assumption above (35). However, MR-Egger regression estimates are less precise if the magnitude of association of all genetic variants with the exposure is similar. In contrast, the weighted median method will provide a consistent estimate only if at least 50% of the weight comes from valid genetic variants, under the condition that no single genetic variant contributes to more than 50% of the weight. Compared with the MR-Egger method, the weighted median method allows the MR assumptions to be violated in a more generalized fashion, for the invalid genetic variants (28). Therefore, although a non-significant estimate was observed using the MR-Egger method, we believe that the rest of the data support a causal effect of platelet count on hypertension.

There are some limitations in this study. As only baseline platelet count is available in TWB, this could cause less robustness, leading to an error of estimation due to individual variability, and should be compensated for by the averaging of measurements such as mean platelet volume (MPV), which is an indicator of platelet activation and has been reported to be elevated in hypertensive patients. Unfortunately, MPV isn't available in TWB and therefore, a similar analysis using MPV as the exposure needs to be conducted in an independent Taiwanese cohort to verify the conclusions from this study. Secondly, information such as whether the hypertensive patients were on antihypertensive drugs was also unavailable in TWB. Factors such as this are critical, as antihypertensive drug use may have a confounding effect on the causal effect of platelet count on hypertension. Again, future cohort studies involving hypertensive patients need to be conducted by addressing drug use as a confounding variable in the MR analysis. A total of 2,052 (59.0%) study subjects were categorized as hypertensive based on the self-reported questionnaire. Among the 2,052 self-reported hypertensive patients, 854 also showed abnormal blood pressure from the measurement data. Under such circumstances, some misclassification cannot be ruled out. The TWB dataset is from a cross-sectional study. Due to restrictions imposed by the data, we could only observe one direction of the causal effect. Furthermore, for the MR method, the assumption of independence from confounding factors did not hold in some situations, as there may still be some unknown confounding factors that could affect either platelet count or hypertension, or both, which were not recorded in the TWB data. Therefore, the causality cannot be guaranteed with complete confidence, if unknown factors affect the relationship. The only way to account for and eliminate unknown confounding factors is to reveal them under varying conditions between platelet count and hypertension by conducting plenty of trials with different study designs. According to the exclusion-restriction assumption for MR, the effect of genetic variants on hypertension is exerted through the platelet count only. However, this assumption is difficult to confirm (and to believe). Even though some methods have been developed to solve this problem, such as the weighted median method, there is residual bias that is hard to eliminate, and more domain knowledge and more extensive exploration on the genetic variants are needed. Even though these issues were beyond the scope of our research, we believe the findings of this study have been impacted by them, and they need to be addressed in the future.

This study involved a bi-directional MR analysis of causality between platelet count and hypertension. A significant positive causal effect of platelet count on hypertension was revealed, whereas the causal effect of hypertension on platelet count was reported to be non-significant. Further research on the relationships of other platelet indices with hypertension using larger and multiple data sources can provide more evidence on the relationship between platelet count and hypertension, with the goal of developing better treatments for hypertension.

## Conclusion

Higher platelet count was observed to have a significant causal effect on the elevated risk of hypertension through this bi-directional MR study. However, future functional assays are warranted to elucidate the related biological pathways and pathogenic mechanisms.

## Data Availability Statement

Researchers can obtain the data by request to Taiwan Biobank through the official application. Requests to access these datasets should be directed to Taiwan Biobank team, https://www.twbiobank.org.tw/new_web.

## Ethics Statement

The studies involving human participants were reviewed and approved by Taiwan Biobank. The patients/participants provided their written informed consent to participate in this study.

## Author Contributions

The study was conceptualized and designed by T-PL. Data curation was done by T-HH and C-HL. Data analysis and interpretation were done by P-CC, AC, and M-CW. Resources and supervision were done by T-PL and AC. Writing, reviewing, and editing of the manuscript were done by AC and P-CC. All authors have read and approved the final version of the submitted manuscript.

## Funding

The work was supported in part by the Center of Genomic and Precision Medicine, National Taiwan University, Taiwan (106R8400); the Center for Biotechnology, National Taiwan University, Taiwan (GTZ300); the Taiwan Ministry of Science and Technology (Grant Nos. MOST-109-2314-B-002-151-MY3 and MOST-106-2314-B-002-134-MY2); and the National Taiwan University Higher Education Sprout Project (NTU-110L8810) within the framework of the Higher Education Sprout Project by the Ministry of Education (MOE) in Taiwan. The funders had no role in the design and conduct of the study, collection, management, analysis and interpretation of the data, preparation, review, or approval of the manuscript, and decision to submit the manuscript for publication.

## Conflict of Interest

The authors declare that the research was conducted in the absence of any commercial or financial relationships that could be construed as a potential conflict of interest.

## Publisher's Note

All claims expressed in this article are solely those of the authors and do not necessarily represent those of their affiliated organizations, or those of the publisher, the editors and the reviewers. Any product that may be evaluated in this article, or claim that may be made by its manufacturer, is not guaranteed or endorsed by the publisher.
