# Supplementary material for: Elucidation of a Causal Relationship Between Platelet Count and Hypertension: A Bi-Directional Mendelian Randomization Study
Source: Front Cardiovasc Med. 2021 Nov 26;8:743075. doi: 10.3389/fcvm.2021.743075 (PMC8661012; doi:10.3389/fcvm.2021.743075)
Supplement: Supplementary file 1 [file Data_Sheet_1.docx]

Supplementary Material

Elucidation of a causal relationship between platelet count and hypertension: a bi-directional Mendelian Randomization study

Po-Chun Chiu^1,†^, Amrita Chattopadhyay^2,†^, Meng-Chun Wu^1,†^, Tzu-Hung Hsiao^3^, Ching-Heng Lin^3^, Tzu-Pin Lu^1,2,4^

^1^Department of Public Health, National Taiwan University, Taipei, Taiwan

^2^Bioinformatics and Biostatistics Core, Centre of Genomic and Precision Medicine, National Taiwan University, Taipei 10055, Taiwan

^3^Department of Medical Research, Taichung Veterans General Hospital, Taiwan

^4^Institute of Epidemiology and Preventive Medicine, National Taiwan University, Taipei, Taiwan

^†^Joint First Authors with equal contribution.

*To whom correspondence should be addressed.

Tzu-Pin Lu

Department of Public Health, Institute of Epidemiology and Preventive Medicine, National Taiwan University, Taipei 10055, Taiwan

Phone: +886-2-3366-8042, Fax: +886-2-3322-4179, E-mail: tplu@ntu.edu.tw

# Supplementary Tables

Table S1. Genome-wide association studies with genetic variants related to platelet count.

|  | **Overlapped Gene** | **Date of Publication** | **Sample Size** | **Sample Ethnicity** | **Citation** |
| --- | --- | --- | --- | --- | --- |
| 1. GWAS in Japanese | JAK2,  CABLES1 | 2018-02-05 | n=108,208 | East Asian | Kanai, Akiyama [17] |
| 2. The Allelic Landscape of Human Blood Cell | CABLES1,  DNM3 | 2016-11-17 | n=166,066 | European | Astle, Elding [18] |
| 3. GWAS of platelet in Hispanic or Latin American | JAK2,  HMIP | 2016-01-21 | n=12491 | Hispanic or Latin American | Schick, Jain [19] |
| 4. GWAS in Korean | NA | 2014-12-31 | n=8,842 | East Asian | Oh, Kim [20] |
| 5. GWAS of platelet related | CABLES1 | 2013-09-12 | n=13,582 | European | Gusev, Lee [21] |
| 6. New gene function in platelet formation | CABLES1 | 2011-11-30 | n=48,666 | European | Gieger, Radhakrishnan [22] |
| 7. GWAS in Japanese biochemical traits | CABLES1,  HBS1L | 2012-02-07 | n=14,806 | East Asian | Kamatani, Matsuda [23] |

Table S2. Genome-wide association studies with genetic variants related to hypertension.

|  | **Overlapped Gene** | **Date of Publication** | **Sample Size** | **Sample Ethnicity** | **Citation** |
| --- | --- | --- | --- | --- | --- |
| 1. GWAS study of blood pressure and hypertension | ATP2B1  CASZ1  CYP17A1  SH2B3 | 2009-05-10 | n=29136 | European | Levy, Ehret [24] |
| 2. GWAS study in Chinese identifies novel loci for blood pressure and hypertension | ATP2B1  CASZ1  FGF5  CYP17A1 | 2014-09-23 | n=11816 | Chinese population | Lu, Wang [25] |
| 3. GWAS study identifies L3MBTL4 as a Novel Susceptibility Gene for Hypertension | ATP2B1  CASZ1  FGF5  CYP17A1 | 2016-08-02 | n=16970 | Chinese population | Liu, Hu [26] |
| 4. Trans-ancestry meta-analysis identify rare and common variants associated with blood pressure and hypertension [27] | ATP2B1  CASZ1  FGF5 | 2016-10-01 | n=165276  n=192763 | Europeans  Europeans&  South Asians | Surendran, Drenos [27] |

Figures


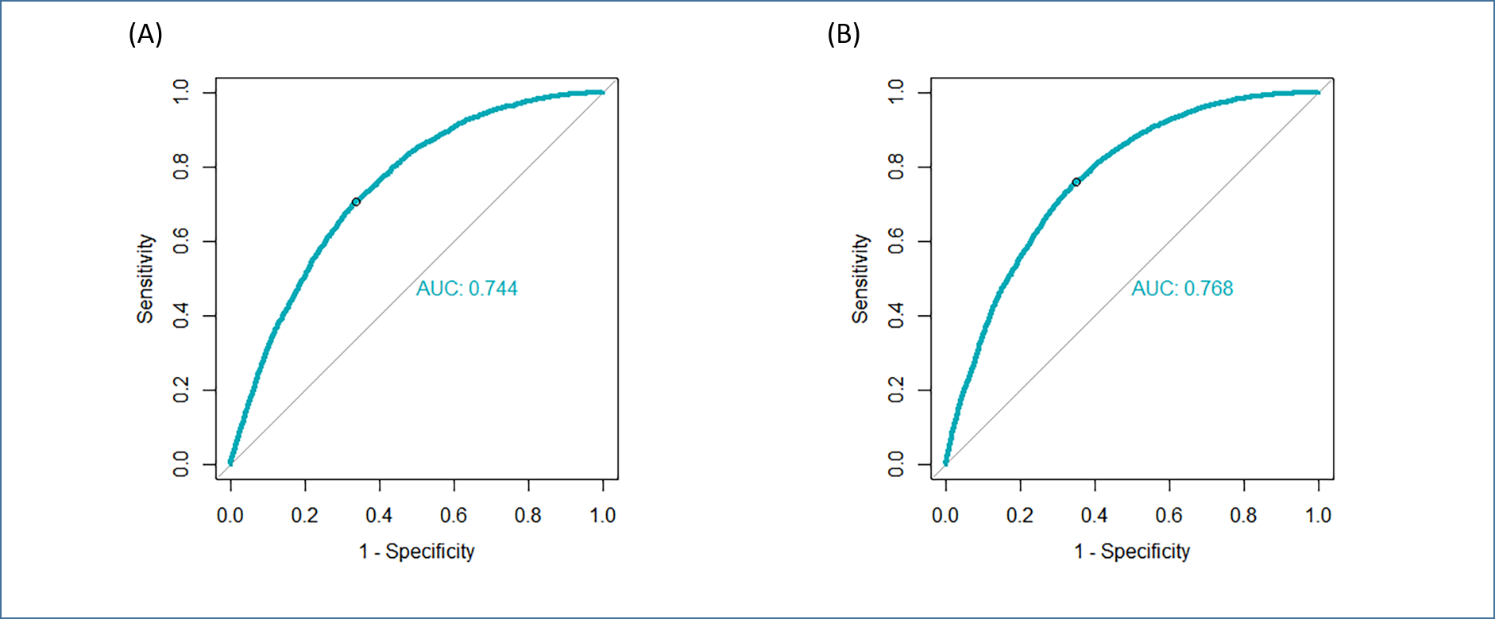


Figure S1. ROC curves for identifying the best cutoff value of platelet count to classify hypertensive and non-hypertensive patients. (A) Age, and sex adjusted model. (B) Multivariate adjusted model, with adjustment variables as age, sex, fasting glucose, hematocrit, triglyceride, high density lipoprotein cholesterol, hemoglobin, red blood cell count, and white blood cell count.


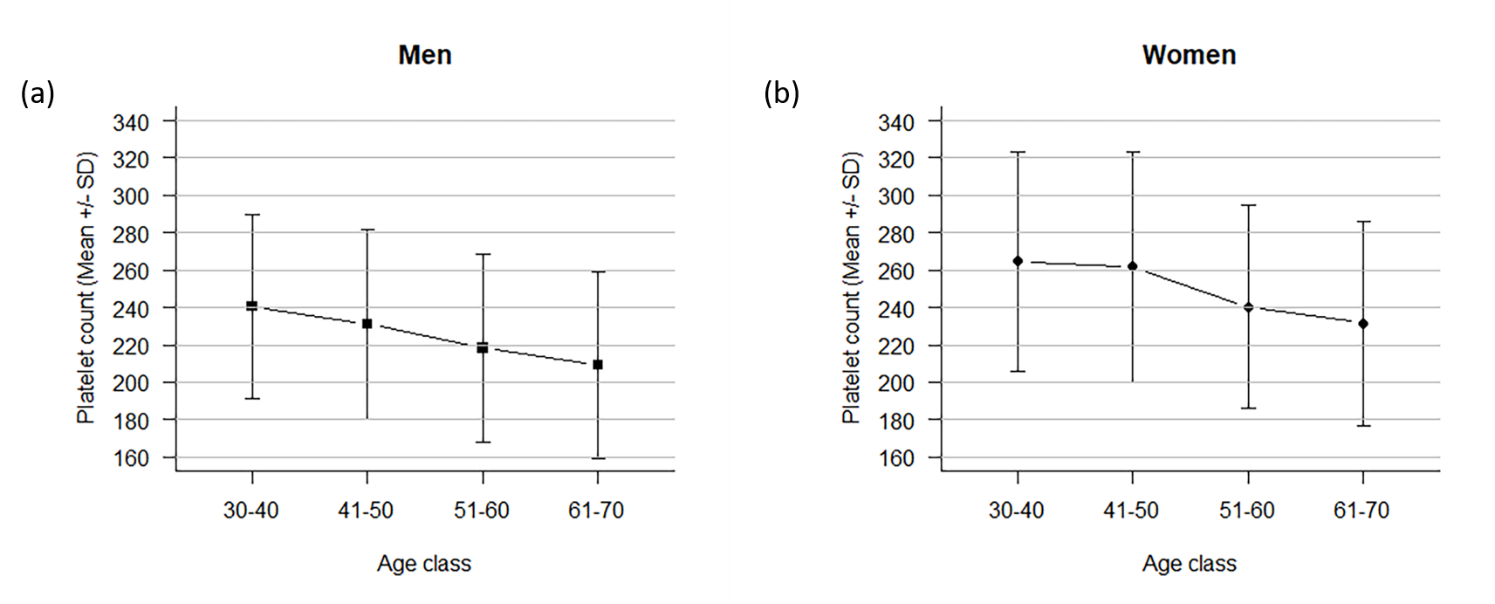


Figure S2. Distribution of platelet count over age for (a) Men and (b) Women
