# Supplementary figures and images for: Elucidation of a Causal Relationship Between Platelet Count and Hypertension: A Bi-Directional Mendelian Randomization Study
Source: Front Cardiovasc Med. 2021 Nov 26;8:743075. doi: 10.3389/fcvm.2021.743075 (PMC8661012; doi:10.3389/fcvm.2021.743075)

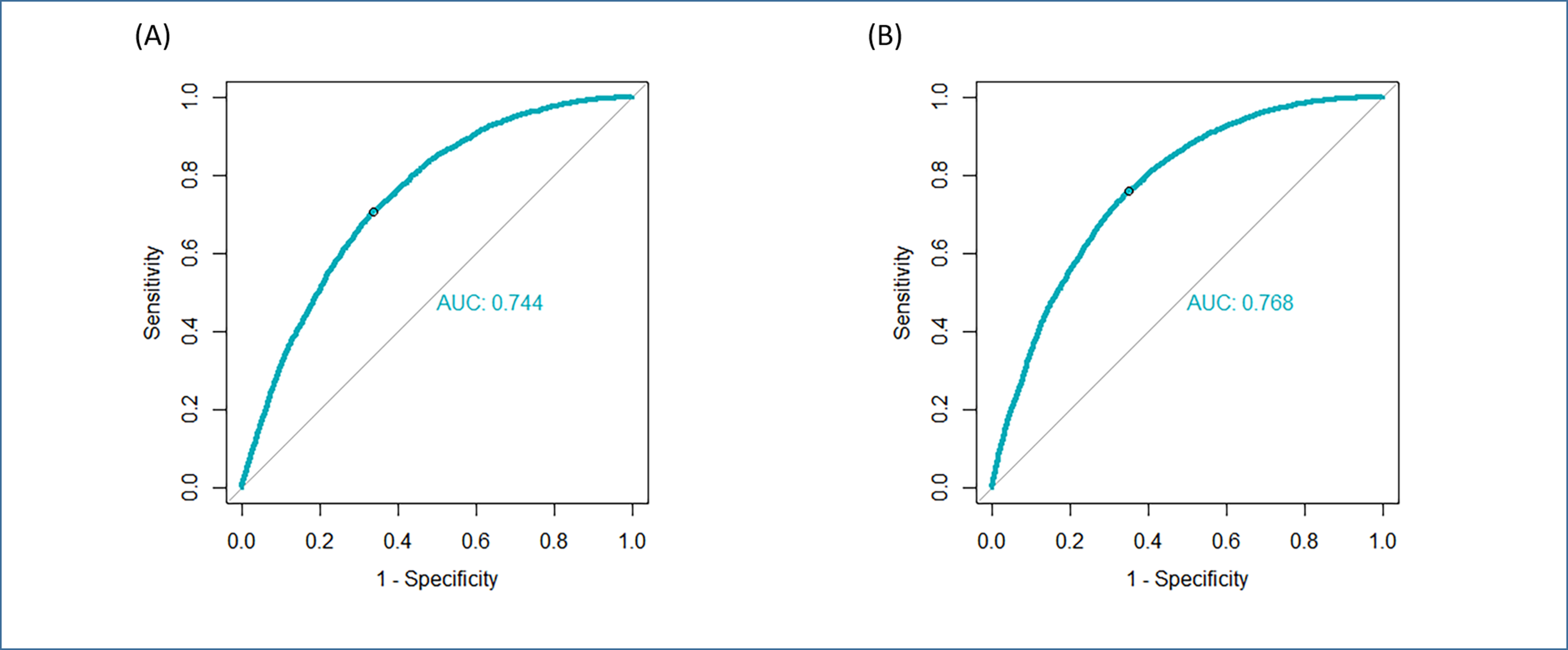

Supplement: Supplementary file 2 [file Image_1.tif]

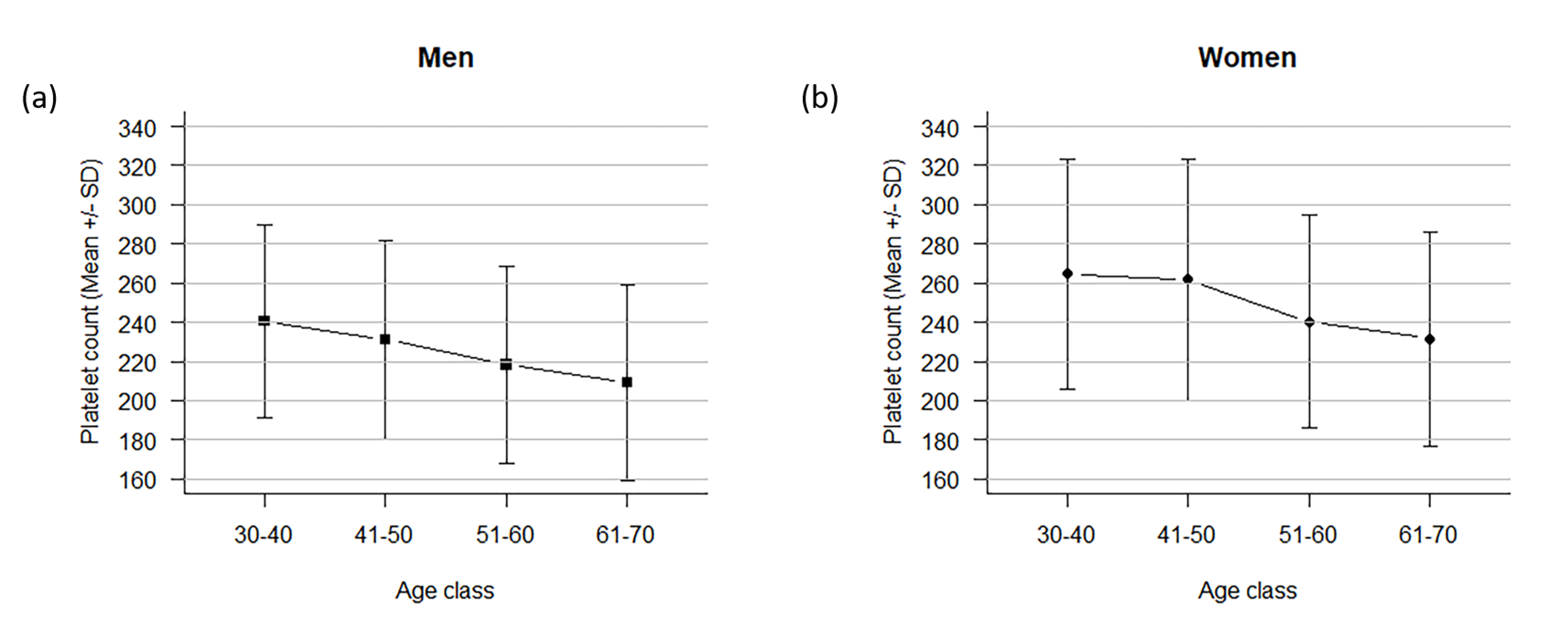

Supplement: Supplementary file 3 [file Image_2.tif]
